# Teaching Deaf learners in multi-grade classes: Perceptions from a rural Mpumalanga special school

**DOI:** 10.4102/ajod.v14i0.1710

**Published:** 2025-10-24

**Authors:** Margaret Chauke, Raesetja G. Ledwaba, Jacomina M. Motitswe

**Affiliations:** 1 Department of Inclusive Education, College of Education, University of South Africa, Pretoria, South Africa

**Keywords:** Deaf learners, multi-grade classrooms, South African Sign Language, asset-based approach, curriculum differentiation, inclusive education

## Abstract

**Background:**

Teaching Deaf learners in multigrade classes contributes to learning challenges and requires implementation of curriculum differentiation. However, limited South African Sign Language (SASL) proficiency and learning resources hinder inclusive education in special schools.

**Objectives:**

This study aimed to explore teachers’ perceptions in supporting Deaf learners in multigrade classrooms, focusing on challenges and teaching strategies for inclusive education.

**Method:**

A qualitative approach with a descriptive phenomenological design was used. Six teachers from a special school were selected using purposive sampling. Data collection involved focus group discussions, classroom observations, document analysis, and field notes. Thematic analysis was employed to generate findings.

**Results:**

Teachers face persistent challenges, including inadequate resources, limited SASL proficiency, and insufficient professional development. To address these challenges, they employ multimodal teaching strategies, advocate for enhanced SASL training, and integrate visual aids to foster inclusive learning.

**Conclusion:**

Teachers used gestures, assistive technologies and visual aids in supporting Deaf learners. The Department of Basic Education should implement structured SASL training, expand access to assistive technologies, and develop an inclusive curriculum tailored to Deaf learners’ needs. Continuous professional development and systematic monitoring are essential for improving teacher effectiveness and promoting inclusive education.

**Contribution:**

This study contributes to the understanding of teacher experiences in supporting Deaf learners in multigrade classes, systemic barriers and adaptive strategies they employ to manage multigrade deaf education. Furthermore, the findings of this study will inform future teacher training on the use of SASL and provide policy recommendations to improve curriculum differentiation.

## Introduction

Special schools are central to the provision of education for learners with disabilities in South Africa, particularly in rural areas where mainstream inclusive education practices often remain under-developed (Donohue & Bornman 2014; Engelbrecht & Green 2018). These schools serve as essential educational spaces for learners with diverse disabilities, including those who are Deaf or hard of hearing. In rural provinces such as Mpumalanga, the situation is further complicated by systemic issues, including low socio-economic background, teacher shortages and inadequate infrastructure (Department of Basic Education [DBE] 2025; Khoaeane & Taylor 2016). One particularly under-researched aspect is how Deaf learners are taught in multi-grade classrooms at special schools. Multi-grade classrooms, common in rural communities, are necessitated by limited resources, fewer schools, staffing constraints and low enrolment numbers (Kivunja & Sims 2015; Taole 2019). This model, established to support the Millennium Development Goals (MDGs) and the Education for All (EFA) objectives, involves teaching learners of varying ages, grades and ability levels within a single classroom (Kivunja & Sims 2015; Taole 2019).

Teaching Deaf learners in multi-grade classrooms presents unique pedagogical and linguistic challenges, particularly in resource-constrained settings like South Africa, where access to specialised schools and support services is limited (Khalid, Singh & Chohan 2024). It demands a high level of adaptable and differentiated strategies, lesson planning complexity and individualised support (Khalid et al. 2024; Naparan & Alinsug 2021). For teachers of Deaf learners, these challenges are further compounded by barriers such as limited proficiency in South African Sign Language (SASL), a shortage of skilled interpreters, and scarce resources (Moustache & Makhoba 2024). Addressing these needs is critical to fostering an inclusive learning environment, particularly in rural areas.

In the rural context of Mpumalanga, where there is only one special school for learners with severe to profound disabilities in a district, Deaf learners from various grades are combined into a single class. This is largely because of the limited number of teachers proficient in SASL. This situation not only undermines the quality of education, but also contradicts the principles of equity and inclusivity embedded in South Africa’s policy on Screening, Identification, Assessment and Support (SIAS) (DBE 2014) and the Education White Paper 6 on Special Needs Education (EWP 6) (Department of Education [DoE] 2001). The SIAS policy emphasises early identification and individualised support, yet the infrastructure and capacity to implement such provisions in rural areas remain severely lacking.

Research has shown that teachers in rural special schools often face challenges such as limited access to professional development in Deaf education and SASL, a lack of interpreter services and inadequate technological support for visual learning (Grace 2023; Naparan & Alinsug 2021). These factors collectively hinder the implementation of inclusive and equitable education for Deaf learners. Moreover, in multi-grade classrooms, the absence of grade-specific learning materials in SASL and the lack of clear policy guidelines further exacerbate the problem (Hlalele 2012; Moustache & Makhoba 2024). In contrast, Wilderman and Nomdo (2007) argue that enhancing teacher support through ongoing professional development in SASL and the provision of visual aids could significantly improve inclusive education in these contexts.

This article explores the lived experiences and perceptions of teachers at a special school in Mpumalanga, examining their daily challenges and strategies for supporting Deaf learners in multi-grade classrooms. By foregrounding teacher voices, the study aims to shed light on both the systemic barriers and the adaptive strategies employed to manage the demands of multi-grade Deaf education in under-resourced environments. Understanding these dynamics is essential for informing future teacher training, curriculum adaptation and differentiation and policy refinement to enhance the educational experiences of Deaf learners in South Africa’s special schools.

### Defining ‘deafness’ and Deafness in an inclusive education context

Deaf education and inclusive education are two interconnected, yet distinct paradigms that significantly shape the educational experiences of learners with hearing impairment. It is important to first provide a definition and distinction between deaf and Deaf. Deaf education has evolved over the past decades. It has shifted from a medical model, which focused on hearing loss and impairment, to a more culturally affirmative perspective that recognises Deaf learners as part of the linguistic and cultural minority (Kusters, De Meulder & O’Brien 2017; Leigh, Andrews & Harris 2018). Deaf learners are seen not merely as individuals with a disability, but as members of the Deaf community, often with their own language, norms and identity (Clark 2025; Leigh et al. 2018). The distinction between deaf (lowercase ‘d’) and Deaf (uppercase ‘D’) is central in this discourse. The term deaf generally refers to the audiological condition of hearing loss and is often associated with individuals who use assistive devices and spoken language, without necessarily identifying with the Deaf community (Inclusive Education n.d.). In contrast, Deaf denotes individuals who identify as part of a cultural and linguistic community that uses sign language such as SASL and shares a collective identity rooted in shared experiences and values (Clark 2025; Kusters et al. 2017).

Understanding these distinctions enables teachers to support learners more effectively by affirming their identities and communication preferences. Whether a learner identifies as deaf or Deaf can influence their participation, peer relationships and engagement in the classroom (Leigh et al. 2018). Acknowledging these differences fosters inclusive, respectful and empowering learning environments.

For Deaf learners, inclusive education remains contested. While the EWP 6 policy (DoE 2001) promotes integration of learners with disabilities into mainstream schools, scholars argue that inclusion should not imply uniformity (Donohue & Bornman 2014; Engelbrecht & Muthukrishna 2019). Instead, the unique linguistic and cultural needs of Deaf learners require differentiated support systems such as the use of SASL, Deaf education specialists, and deaf role models (Storbeck 2018).

### Definition of multi-grade classrooms

Multi-grade classrooms are educational settings in which a single teacher instructs learners from multiple grade levels simultaneously. These environments are particularly prevalent in rural or under-resourced areas where limited enrolment, staffing shortages or geographic isolation make single-grade classrooms impractical (Kalender & Erdem 2021; Makoelle & Malindi 2014). Within the context of Deaf education, multi-grade classrooms introduce specific pedagogical complexities. Teachers must accommodate learners with differing academic levels and varied exposure to SASL, the primary mode of communication. As a result, teachers must implement scaffolded communication techniques and differentiated instruction to support meaningful engagement and learning (eds. Phasha, Mahlo & Dei 2017).

### Challenges of multi-grade classrooms for deaf learners

Teaching in multi-grade classrooms presents significant challenges, particularly when supporting Deaf learners. The diversity of educational needs requires teachers to adapt content and delivery methods, often without the benefit of specialised training in visual communication strategies such as SASL, visual aids and written text (Moustache & Makhoba 2024). Many South African teachers lack sufficient training in SASL, which impairs their ability to engage Deaf learners effectively and provide individualised instruction (Molapisi & Phasha 2024).

Research indicates that Deaf learners in multi-grade settings frequently encounter language barriers because of insufficient teacher preparedness and a scarcity of resources tailored to their communication needs. Inadequate SASL proficiency among educators can limit comprehension and reduce learner participation (Makoelle & Malindi 2014). To address this, teachers must employ a broad range of multimodal and visually oriented teaching strategies. These include the use of captions, subtitles and structured visual scaffolding, which are essential for facilitating understanding and inclusion (Wildeman & Nomdo 2007; Yoon & Kim 2011).

### The asset-based approach in deaf education

An asset-based approach provides a promising framework for enhancing inclusive practices in multi-grade classrooms. This perspective emphasises harnessing teachers’ and learners’ existing strengths, skills and community resources to overcome constraints (Donald, Lazarus & Lolwana 2010). By focusing on what learners and teachers bring to the classroom rather than on their deficits, an asset-based approach fosters a sense of empowerment and adaptability among teachers. For teachers teaching Deaf learners, this includes drawing on the visual learning strengths of Deaf learners and incorporating community resources, such as local SASL experts, to support language acquisition and comprehension (Ngobeni, Maimane & Rankhumise 2020).

Research shows that teachers who adopt an asset-based approach are more resilient and better equipped to address the challenges of multi-grade teaching (Eloff, van der Merwe & Karsten 2024; Makoelle & Malindi 2014; Mouton 2012). This approach encourages collaboration and resource-sharing in multi-grade classrooms, helping teachers use available tools to create more inclusive and effective learning environments. Phasha et al. (2017) argue that, particularly in rural and under-resourced schools, community involvement and resource-sharing are essential to support teachers in meeting the diverse needs of Deaf learners.

### Multimodal teaching strategies

Multimodal teaching strategies are widely advocated as a solution for engaging Deaf learners in multi-grade settings. These strategies include using visual aids, written instructions, gestures and peer-assisted learning, which accommodate the needs of learners with varying levels of understanding (Grace 2023). Research indicates that multimodal approaches enhance comprehension and increase engagement and motivation among Deaf learners (Francisco & Padilla 2024; Weber et al. 2024). Molapisi and Phasha (2024) found that integrating visual and gestural forms of communication alongside SASL can bridge communication gaps and support effective learning for Deaf learners.

### Policy and support for inclusive deaf education

While South Africa’s policy on SIAS aims to support inclusivity, it often fails to address the specific needs of Deaf learners in multi-grade classrooms (DBE 2014). Current policies lack provisions for the specialised training and resources that teachers need to support Deaf learners effectively. Wildeman and Nomdo (2020) argue that policies must be tailored to address the unique needs of multi-grade and special education settings. Studies recommend that professional development programmes include SASL training and equip teachers with multimodal teaching techniques to bridge the resource gap in special schools for Deaf learners (Moustache & Makhoba 2024).

Policy reform that addresses the inclusion of Deaf learners in multi-grade classrooms would involve funding for SASL training and providing visual aids and other teaching resources. Targeted professional development and curriculum differentiation are crucial for empowering teachers and creating accessible educational environments for all learners, regardless of their abilities (Phasha et al. 2017). Without systemic support, teachers face significant barriers that impact their ability to deliver inclusive education to Deaf learners, limiting the potential for equitable access to quality education.

The literature highlights the pressing need for systemic support and targeted strategies to enhance inclusive education for Deaf learners in multi-grade classrooms. Effective implementation of multimodal teaching, professional development in SASL and tailored resources are essential steps to improve educational outcomes for Deaf learners (Mapepa & Magano 2018). Addressing these challenges will contribute to the broader goals of inclusive education, allowing Deaf learners to thrive in resource-limited settings. Educational stakeholders can foster more inclusive and supportive learning environments by supporting teachers in implementing multimodal teaching strategies to address the needs of Deaf learners effectively (Mpu & Adu 2021).

### Theoretical framework

The current study employs an asset-based approach to examine teachers’ experiences in addressing the educational needs of Deaf learners in multi-grade classrooms. Originally introduced by Kretzmann and McKnight (1993), this approach shifts the focus from deficits to strengths, emphasising the knowledge, skills and resources that teachers and learners bring to the classroom. In this framework, diversity is seen as a resource rather than a barrier, encouraging teachers to recognise and build upon learners’ inherent strengths and community resources.

In multi-grade classrooms, where learners of varying abilities and ages learn together, the asset-based approach fosters resilience and adaptability among teachers (Taole 2019). Teachers rely on innovative, flexible strategies to support inclusive learning despite resource limitations (Makoelle & Malindi 2014). These strategies include multimodal teaching methods such as visual aids, captions, subtitles, gestures and interactive materials, which enhance accessibility and cater to the learning preferences of Deaf learners (Yoon & Kim 2011).

Community collaboration is integral to this approach, particularly in under-resourced areas where local experts and parents can support teachers and learners, especially in SASL development (Ngobeni et al. 2020). Fluent SASL users within the community contribute to language acquisition, enriching the educational experience for Deaf learners while strengthening school–community ties. The asset-based framework also acknowledges the unique abilities of Deaf learners, such as enhanced visual-spatial skills, which can be leveraged through visually oriented teaching practices (Moustache & Makhoba 2024). By adapting instructional methods to harness these strengths, teachers create inclusive and personalised learning experiences that support all learners.

Teachers in resource-limited settings demonstrate adaptability, resilience and dedication, positioning themselves as valuable assets in inclusive education (Engelbrecht & Muthukrishna 2019; Wabule 2020). Many develop SASL proficiency to meet learners’ needs, embodying the commitment to inclusive teaching (Weber et al. 2025). Professional development plays a crucial role in enhancing teacher efficacy, particularly in SASL and multimodal teaching strategies. Husum and Waseem (2024) highlight how targeted training significantly improves teachers’ ability to support diverse learners, ensuring sustainable inclusion.

Furthermore, community engagement and resource-sharing reinforce the long-term success of inclusive education. Collaborative networks, involving families, local organisations and peer teachers, create sustainable support systems that cater to diverse learner needs (Phasha et al. 2017). These networks are particularly crucial in multi-grade classrooms, where individualised support varies widely.

Ultimately, the asset-based approach provides a holistic and empowering framework for inclusive education. By focusing on the strengths of both teachers and learners, it promotes collaboration, resilience and sustainable inclusion. This model not only addresses challenges but also harnesses existing classroom and community assets to foster educational success for all learners.

### Rationale

Supporting learners who require additional assistance, such as those who are Deaf, is essential to foster effective learning. The Department of Basic Education (DBE 2014) emphasises the importance of facilitating the learning of Deaf learners and recommending the use of SASL interpreters to assist classroom teachers lacking SASL proficiency. Furthermore, trained teachers specialising in SASL must provide accessible and meaningful instruction. Deaf learners in multi-grade classrooms face unique challenges, particularly in resource-constrained settings like South Africa. In rural areas, such as the province of Mpumalanga, teachers face severe resource limitations, lack specialised training and receive limited support in educating Deaf learners.

While policies like the SASL framework promote inclusive education in South Africa, they fall short of addressing the unique needs of Deaf learners in multi-grade settings (DBE 2014). Many teachers have limited SASL training, and educational resources specifically tailored for Deaf learners are scarce, particularly in rural areas (Shawula 2023). These policy and resource gaps highlight the need for studies examining how teachers can receive better support to ensure quality education for Deaf learners in multi-grade classrooms.

This study is anchored in the asset-based approach, emphasising the strengths and resources teachers, learners and communities bring to the educational context. In contrast to deficit models that focus on limitations, the asset-based approach values adaptability, resilience and collaboration as key elements of inclusive education (Husum & Waseem 2024). By investigating how teachers harness available resources and skills to support Deaf learners, the current study seeks to contribute a more complex understanding of effective, context-specific teaching strategies.

This study is significant in several ways. It addresses the limited body of literature on the educational experiences of Deaf learners in multi-grade classrooms in rural South Africa, a setting often under-represented in educational research (Suubi 2013). Additionally, the study aims to provide evidence-based recommendations for the DBE to guide the development of inclusive policies and teacher-training programmes. By highlighting the importance of multimodal teaching strategies, SASL training and community involvement, this study offers practical insights for fostering inclusive learning environments that empower teachers of Deaf learners.

Therefore, this study addresses a significant gap in the literature by exploring the specific educational needs and potential interventions for Deaf learners in multi-grade classrooms, particularly within rural and resource-constrained contexts. While inclusive education policies have gained momentum across sub-Saharan Africa, the unique challenges faced by Deaf learners in multi-grade classrooms remain under-examined (Mapepa & Magano 2018). Adopting an asset-based approach, this study shifts the focus from deficits to the strengths, capacities and local knowledge embedded within rural communities and schools (Kretzmann & McKnight 1993). Through a comprehensive literature review, it identifies how community assets such as the skills of teachers, the resilience of learners and the support of local networks can be mobilised to create more inclusive and responsive learning environments.

Teachers are central to this inquiry, not merely as implementers of policy but as co-creators of contextually relevant solutions. Their lived experiences and adaptive strategies offer valuable insights into how existing resources can be leveraged to support Deaf learners in under-resourced settings (Ndwandwe & Joseph 2025). By integrating empirical evidence and teacher narratives, this study aims to inform teachers, policymakers and stakeholders on how to enhance educational practices, policies and support systems through a strengths-based, community-driven lens that aligns with the principles of asset-based development.

In fulfilling these objectives, the study aims to contribute meaningful insights into the development of more inclusive, responsive and effective educational environments for Deaf learners in multi-grade settings. By centring the voices of teachers and examining existing practices, this research seeks to inform future policy, teacher training and classroom strategies that promote equitable learning opportunities for all.

Guided by these inquiries, the study aimed to understand teachers’ perceptions of supporting Deaf learners in multi-grade classrooms. It also identified and analysed the educational needs of Deaf learners and investigated the use of multimodal teaching strategies. Finally, the study explored how both challenges and existing support structures influence the inclusivity of multi-grade classrooms, as reflected in the lived experiences of the participating teachers.

## Research design and methodology

This study employed a qualitative research design grounded in the interpretive research paradigm, which posits that reality is constructed through social interactions and individual experiences (Guba & Lincoln 2005). This paradigm was particularly appropriate for investigating how teachers understand and implement support services for Deaf learners in multi-grade rural classrooms. Central to this design was the intention to privilege teacher voices, recognising them as knowledge holders whose experiences offer invaluable insights into the practices, barriers and innovations shaping inclusive education (Babbie & Mouton 2020; Creswell & Plano Clark 2017).

The interpretive lens was operationalised through a descriptive phenomenological approach, which allowed the research to explore the lived experiences of teachers working in a special school context in Mpumalanga, South Africa. Phenomenology was suited to unearthing the structural features of experience by allowing participants to articulate challenges and strategies in their own words (Khan 2014; Smith, Larkin & Flowers 2021).

### Research site and participants

The research was conducted at a special school in Mpumalanga province, South Africa, which caters to Deaf learners within multi-grade classrooms. The school is situated in a resource-constrained rural area, characterised by limited access to assistive technologies and a shortage of formally trained professionals in Deaf education. Participants were selected using non-probability convenience sampling and included teachers with experience in teaching Deaf learners across multiple grade levels. Their direct involvement in inclusive practices within these contexts ensured the relevance and richness of their contributions.

The study sample consisted of six female teachers: one departmental head and five level-one teachers, including one who is Deaf. The participants’ ages ranged from 30 years to 40 years, with an average age of 45 years. To maintain confidentiality, each participant was given a pseudonym, from Teacher 1 to Teacher 6. All participants held formal teaching qualifications, with the departmental head also possessing an Honours degree in educational management. Notably, four of the level-one teachers had completed Advanced Certificates in Inclusive Education (ACE), while one held a Postgraduate Diploma in the same field (see Table 1). This specialised training aligns with existing literature, suggesting that advanced qualifications in inclusive education enhance teachers’ abilities to implement multimodal teaching strategies tailored to the diverse needs of learners (Makoelle 2021; Uys & Hugo 2022). Such qualifications are crucial in fostering inclusive practices, enabling teachers to effectively address various learning needs.

**TABLE 1:** Background information of participants.

Teachers	Gender	Age (years)	Qualifications	Major subjects
T1	F	45	B.Ed. Honours in Educational Management	English and Mathematics
T2	F	35	Post Graduate Diploma in Inclusive Education	South African Sign Language
T3	F	42	Advanced Certificate in Inclusive Education	English and Life Skills
T4	F	35	Advanced Certificate in Inclusive Education	English and Siswati
T5	F	37	Advanced Certificate in Inclusive Education	English and Natural Sciences
T6	F	40	Advanced Certificate in Inclusive Education	English, Mathematics and SiSwati

Note: Participants were assigned numerical codes from T1 to T6. ‘T’ for teachers.

### Data collection

Data were gathered using focus group discussions (FGDs), classroom observations and document analysis, enabling data triangulation and increasing the credibility of the findings (Creswell 2014).

### Focus group discussions

Focus group discussions served as the principal method for exploring teachers’ insights, allowing for dynamic discussions on shared experiences, instructional challenges and multimodal strategies. Teachers provided detailed accounts of their efforts to adapt to Deaf learners’ needs using tools like SASL, visual aids and peer scaffolding (Moustache & Makhoba 2024). Follow-up questions encouraged clarification and deepened exploration, particularly around systemic challenges, learner engagement and support mechanisms.

### Classroom observations

Classroom observations provided real-time insight into pedagogical practices and inclusive strategies used in multi-grade settings. These included teachers’ use of SASL, visual prompts and differentiated instruction to support learner comprehension. Observations revealed how teachers navigated limitations such as insufficient resources, while also showcasing adaptive behaviours that maintained learner engagement and participation (Moustache & Makhoba 2024).

### Document analysis

A review of curriculum materials, institutional records and policy documents helped contextualise the teaching environment. This method identified resource gaps and illustrated how national education frameworks either supported or constrained inclusive teaching. The analysis highlighted the interplay between formal policy and actual practice, revealing inconsistencies in policy implementation and opportunities for reform (Cardno 2018; DBE 2014).

### Interview schedule

The interview schedule guiding FGDs was developed through initial consultations with teachers and a contextual needs analysis (Mapepa & Magano 2018). The schedule covered five key themes:

- Rapport-building: Exploring teachers’ backgrounds and motivations (Ndwandwe & Joseph 2025).
- Educational needs and teaching strategies: Identifying challenges and effective pedagogical responses (Storbeck 2018).
- Multimodal approaches: Investigating the use of visual, gestural and digital aids (Hauser et al. 2010).
- Learner engagement: Understanding strategies for fostering inclusion and peer interaction (Humphries et al. 2013).
- Systemic barriers and supports: Examining institutional constraints and professional development opportunities (Mapepa & Magano 2018).

This structure ensured data were collected in a focused, yet flexible manner, responsive to the nuances of teacher experience.

### Data analysis

Data from FGDs, observations and documents were subjected to thematic analysis, guided by the principles of qualitative coding and interpretive meaning-making (Creswell & Creswell 2022; Ningi 2022). The analytical process was inductive, with patterns emerging organically through careful review and categorisation. Transcripts and field notes were initially open-coded, and themes were developed through iterative cross-referencing and refinement. This ensured findings were grounded in participants’ lived realities, maintaining the phenomenological commitment to experience-centred knowledge (Smith et al. 2021).

### Ensuring scientific rigour: Lincoln and Guba’s trustworthiness framework

The study adhered to Lincoln and Guba’s (1985) framework for establishing trustworthiness in qualitative research:

- Credibility: Achieved through triangulation across data sources, member-checking during FGDs and prolonged engagement with the research site.
- Transferability: Enabled by rich contextual descriptions of the school environment, teacher demographics and observed practices.
- Dependability: Maintained through transparent documentation of methodological decisions, coding frameworks and audit trails.
- Confirmability: Supported by reflexive journaling and the use of external documents to substantiate participant perspectives.

This framework ensured that the findings were not only methodologically sound but also analytically rigorous and ethically grounded.

### Ethical considerations

Ethical clearance was obtained from the University of South Africa Research Ethics Committee on 11 May 2025 (Ref: 2019/05/15/90255194/32MC). Permission was also granted by the Mpumalanga Department of Education and the school principal. The study adhered to ethical guidelines by ensuring the confidentiality of participants, obtaining informed consent, and protecting their well-being throughout the research process. Participants were made aware of their right to withdraw from the study at any time. Additionally, measures were taken to anonymise all identifying information in the reporting of the study (Creswell & Creswell 2022; McMillan & Schumacher 2014).

### Supporting Deaf learners using bilingual-bicultural and multimodal teaching strategies

The school supports a diverse group of learners with various disabilities, including autism, deafness, learning disabilities, moderate to severe intellectual disabilities, multiple disabilities and severe disabilities. To meet these learners’ communication and learning needs, multimodal teaching strategies and specialised support, such as SASL, are crucial. Research demonstrates that multimodal approaches incorporating visual, auditory and kinaesthetic elements enhance learning accessibility and engagement, especially for Deaf learners who benefit from visual and hands-on support (Matolo & Rambuda 2022).

Including a Deaf teacher, who also serves as a counsellor, adds significant value to the school’s bilingual-bicultural model of instruction. This approach enables learners to access academic content and cultural understanding through SASL, fostering cognitive and social inclusion. Deaf teachers play a vital role in promoting language development, cultural identity and a sense of belonging among Deaf learners, which is essential for their psychosocial well-being (Smith et al. 2021). Research highlights the importance of inclusive teaching practices and well-trained, diverse teaching staff in meeting the educational needs of Deaf learners in special education settings (Leigh & Crowe 2020).

## Results

The findings of this study were derived from FGDs, classroom observations and document analysis to highlight the distinct educational needs of Deaf learners in multi-grade classrooms and the strategies teachers employ to address these needs. The following five key themes emerged from the data.

### Theme 1: Distinct educational needs of deaf learners in multi-grade classrooms

Participants highlighted the distinct educational needs of Deaf learners in multi-grade classrooms, particularly in communication support and individualised instruction. These needs are assessed based on language acquisition challenges and comprehension levels, which differ significantly from those of hearing learners. Teacher 1 noted that:

‘Deaf learners struggle with language acquisition and comprehension, requiring tailored teaching strategies. The complexity increases in multi-grade classrooms, where learners have varying academic and developmental levels.’ (T1)

Teacher 2, who is Deaf, stressed the ‘need for extra time and one-on-one support for complex topics, though limited resources in township special schools pose challenges’. Teachers 3, 4, 5, and 6 highlighted the importance of multimodal teaching strategies, including individualised learning plans and enhanced visual aids, but emphasised the need for teacher training to implement these strategies effectively.

### Theme 2: The utilisation of augmentative and alternative communication and South African Sign Language

Regarding the question, how are the augmentative and alternative communication (AAC) methods, such as SASL and other visual and gestural languages, utilised to enhance learning and facilitate communication for Deaf learners? Teacher 2, a deaf school counsellor, mentioned that:

‘South African Sign Language (SASL) is essential for promoting communication, inclusion, and effective classroom participation. She further emphasised that SASL enables Deaf learners to access the curriculum and engage meaningfully in learning activities.’ (T2)

However, Teachers 1, 3, 4, 5, and 6 reported a lack of proficiency in SASL, which poses communication barriers and hinders them from effectively teaching learners with hearing impairments. These participants expressed the need for professional development in SASL to enhance inclusive education practices. Teacher 1 indicated that teachers should employ various AAC methods, such as visual aids, written instructions and gestures, to support learning. Teacher 1 further highlighted the importance of culturally responsive facilitators, including SASL-proficient interpreters, in fostering self-expression and confidence for Deaf learners. Teachers 4, 5, and 6 agreed that visual resources, like picture cards and illustrated storybooks, effectively support vocabulary and concept comprehension.

### Theme 3: The use of multimodal teaching strategies

To address the unique educational needs of Deaf learners effectively, Teachers 1 and 2 reported using diverse multimodal instructional strategies that integrate visual, kinaesthetic and interactive elements, which enhance comprehension and engagement by harnessing multiple sensory channels. They further emphasised that ‘visual aids, such as color-coded tasks and graphic organisers (e.g. story maps and character charts), enabled learners to navigate tasks, build literacy and narrative comprehension skills independently’. The study revealed that technology, including captioned videos and educational applications, provided accessible and interactive content tailored to diverse learning styles (Cawthon & Garberoglio 2017; Marschark & Knoors 2022). Teachers 3, 4, 5, and 6 indicated that hands-on activities, such as using tangible objects in mathematics, facilitated understanding of abstract concepts and promoted direct engagement in multi-grade classrooms with diverse academic levels. Teachers further indicated that they utilised peer-assisted learning, group work, gestures and written instructions to foster an inclusive and active learning environment, supporting cognitive development and inclusivity.

### Theme 4: Active engagement of deaf learners in classroom activities

In response to the question of why Deaf learners need to engage actively in classroom activities and how their participation is promoted, Teacher 2, who is Deaf and serves as a school counsellor, indicated that ‘active engagement is essential for Deaf learners as it fosters meaningful participation, social interaction, and cognitive development in classroom activities’. Teachers 3, 4, 5, and 6 emphasised the need for an inclusive environment that encourages active involvement through visual and interactive methods; this view was aligned with the research conducted by Moustache and Makhoba (2024). Teachers 1 and 2 indicated that the ‘key strategies to promote participation included group discussions, hands-on activities, and peer support systems to build confidence and facilitate communication’. Teachers 3, 4, 5, and 6 highlighted that the challenges, such as limited proficiency in SASL and communication barriers, often hindered engagement, particularly during group activities. To address this, teachers relied on the services of the school counsellor in group settings to bridge communication gaps, enhancing collaboration and reducing isolation, because the school does not have skilled SASL interpreters.

### Theme 5: The need for professional development and institutional support

The findings indicate that teachers in multi-grade classrooms with Deaf learners require continuous professional development and stronger institutional backing to effectively implement inclusive teaching practices. Teacher 4 mentioned that ‘ongoing professional development and institutional support are vital for enhancing inclusive teaching practices for Deaf learners, especially in under-resourced classrooms’. Teachers 3 and 5 also expressed concerns about limited access to essential resources such as learning aids and visual materials, which hinder their ability to meet learners’ diverse needs. They emphasised the importance of sustained training in SASL, multimodal teaching strategies and collaboration with specialists to address these gaps. All participants reported feeling underprepared because of limited training, highlighting the need for institutional initiatives that provide both capacity-building opportunities and the necessary teaching resources. Institutional support through learning aids, policy guidance and access to inclusive education experts was viewed as crucial in enabling teachers to support Deaf learners effectively, despite resource constraints.

## Discussion

This study explored teachers’ experiences in addressing the educational needs of Deaf learners in multi-grade classrooms within a rural special school in Mpumalanga, South Africa. The findings reveal that Deaf learners in these contexts face distinctive educational challenges, particularly related to language acquisition and comprehension, which differ significantly from those of their hearing peers and learners with other disabilities (Molala & Mokala 2025). These challenges are compounded by the multi-grade structure, which merges learners of varying ages, academic levels and developmental stages, requiring teachers to implement differentiated and inclusive teaching strategies (PMG 2025).

A central issue identified was the critical role of communication in facilitating inclusive education. SASL, now officially recognised as the 12th national language, was viewed by participants as essential for enabling Deaf learners to access the curriculum and engage meaningfully in classroom activities (Free State Department of Education 2025). However, many teachers reported limited proficiency in SASL, which hindered effective communication and instruction. This gap also restricted learners’ social interaction and participation. In response, teachers employed a range of AAC strategies, including visual aids, gestures, body language and written instructions, to bridge communication barriers (Gómez-Asencio et al. 2025; Kuyler et al. 2025). Despite these efforts, participants emphasised the urgent need for structured SASL training and access to SASL-proficient interpreters to support classroom learning (DEAFinition 2025).

In the absence of sufficient SASL skills and formal resources, teachers turned to multimodal teaching strategies to support learner understanding and participation. These included visual, tactile and kinaesthetic elements such as captioned videos, graphic organisers and concrete materials for mathematics. Such strategies were particularly effective in multi-grade settings, where learners’ academic abilities vary widely (Deaf Unity 2020; Weber et al. 2024). Teachers also promoted peer-assisted learning and collaborative group work to foster inclusive interactions. However, they noted that these methods require careful facilitation, especially when communication barriers limit peer engagement (Molala & Mokala 2025).

Active learner participation emerged as a cornerstone of inclusive education. Teachers consistently emphasised the importance of involving Deaf learners in classroom activities to promote cognitive development, self-confidence and social integration. Hands-on learning and opportunities for interaction were found to increase motivation and reduce isolation (Böhringer & Storbeck 2025). Yet, these efforts were often constrained by the lack of SASL interpreters and trained personnel to support communication during group tasks. In such cases, school counsellors played a vital role in bridging communication gaps, although their capacity was limited (Isaacs et al. 2025).

Finally, the findings highlight the urgent need for professional development and institutional support to sustain inclusive education in under-resourced environments. Teachers reported feeling inadequately prepared to meet the needs of Deaf learners because of limited training, insufficient resources and the absence of specialist support. They identified continuous SASL training, exposure to inclusive pedagogies and access to assistive technologies and visual materials as priorities for improving teaching effectiveness (DBE 2025). Institutional backing through policy implementation, collaboration with inclusive education experts and systematic resource allocation were deemed essential to strengthen teaching practices and ensure that Deaf learners in multi-grade classrooms are not left behind (Isaacs et al. 2025).

Combined, the findings of this study highlight the complexities of teaching Deaf learners in multi-grade classrooms and the creative, though often constrained, strategies teachers adopt in response. While these teachers demonstrate resilience and innovation, their efforts must be supported through systemic interventions aimed at strengthening inclusive education frameworks, particularly in rural and special school contexts (Isaacs et al. 2025; Molala & Mokala 2025).

### Contributions of the study

This study contributes valuable insights into the lived experiences of teachers supporting Deaf learners in multi-grade classrooms within under-resourced rural special schools in South Africa. By foregrounding teachers’ voices, the study deepens understanding of the contextual challenges that hinder inclusive education, specifically, inadequate SASL proficiency, limited access to teaching resources and the absence of professional development opportunities. It highlights the innovative use of multimodal strategies and AAC methods as adaptive responses to these challenges. The study further adds to the discourse on inclusive education by promoting an asset-based approach, showing how teachers build on learners’ strengths despite systemic limitations. These findings provide an empirical foundation for shaping teacher training, resource allocation and policy interventions aimed at improving the quality of education for Deaf learners in multi-grade classrooms.

### Limitations of the study

While the study offers important perspectives, it has several limitations. Firstly, the research was conducted in a single special school in a rural area of Mpumalanga, which may limit the generalisability of the findings to other contexts, such as urban or mainstream schools. Secondly, the study relied on a small sample of teachers selected through non-probability convenience sampling, which may not fully capture the diversity of experiences across different regions or school types. Thirdly, because of resource constraints, the study did not include perspectives from Deaf learners or parents, which could have enriched the findings by incorporating a broader understanding of the educational experience.

### Recommendations

Based on the findings, several recommendations are proposed to enhance inclusive education for Deaf learners in multi-grade classrooms:

- Structured SASL training: The DBE should implement mandatory and continuous SASL training programmes for all teachers working with Deaf learners, with particular focus on rural and under-resourced schools.
- Provision of teaching resources: Schools must be equipped with visual learning materials, assistive technologies and AAC tools to support differentiated instruction and multimodal learning.
- Deployment of SASL-proficient personnel: Efforts should be made to appoint qualified SASL interpreters or facilitators in schools to bridge communication gaps and promote active learner engagement.
- Curriculum differentiation support: Teachers should receive training in curriculum adaptation strategies that are suited to multi-grade and inclusive settings, enabling them to address varying learner needs effectively.
- Collaboration with specialists: Schools should facilitate partnerships with inclusive education specialists, speech and language therapists and counsellors to support the holistic development of Deaf learners.
- Policy monitoring and implementation: Government and education stakeholders should strengthen monitoring systems to ensure that inclusive education policies are effectively implemented at the school level.

## Conclusion

This study highlighted the multifaceted challenges and adaptive practices involved in teaching Deaf learners in multi-grade classrooms within a rural special school. Teachers demonstrated commitment to inclusion using multimodal strategies, visual aids and AAC methods despite limitations in SASL proficiency and access to resources. However, these efforts are not sustainable without systemic support. Strengthening inclusive education requires investment in teacher development, communication support, curriculum adaptation and inclusive infrastructure. A coordinated, policy-driven response grounded in an asset-based framework is essential for ensuring that Deaf learners are meaningfully included and supported in South African classrooms, particularly in rural and special school contexts.
